# Subcutaneous Punctures

**Published:** 1845-11

**Authors:** 


*Subcutaneous Puncture.*—M. Blandin often employs the subcutaneous method of M. Guérin, in opening scrofulous abscesses and abscesses from congestion—a process which consists, as is already known, in making a puncture at the base of a fold of the skin, at some distance from the abscess, with a flattened trocar, and in withdrawing the pus by means of a *sèringue à aspiration*, adapted to the canula of the trocar. The following is the last case which occurred in M. Blandin's practice:—The disease was an encysted scrofulous abscess in the armpit. The abscess was entirely emptied, and the usual dressing for subcutaneous wounds was afterwards applied. The next and the following days affairs went on as they usually do in such a case—that is to say, without the occurrence of any annoying symptom; the little wound made by the puncture immediately reunited, the tumour had disappeared, and the patient did not complain of the slightest pain. After the lapse of a fortnight the abscess reappeared, but was of less size than before the operation. It was evacuated again in the same manner, and with the same results. Some time afterwards the abscess recurred, and was punctured for the third time, with an equal immunity from annoying symptoms, but was followed this time with complete efficacy--that is to say, that, after the third evacuation, the tumour totally disappeared, the cyst collapsed, and was not reproduced. In order to favour its resolution, mercurial frictions were practised after each operation. M. Blandin considers this plan of exceeding utility in those cases in which it is capable of application; and also that it is applicable in all cases of scrofulous abscesses.—*London Medical Times, from Gazette des Hôpitaux.*

